# Frequency, types, and factors associated with complementary and alternative medicine use among patients on maintenance haemodialysis

**DOI:** 10.1186/s12906-022-03815-7

**Published:** 2022-12-07

**Authors:** Alex Tatang Mambap, Gwendoline Enda Ukum, Denis G. Teuwafeu, Mahamat Maimouna, Gloria Enow Ashuntantang

**Affiliations:** 1 Department of Clinical Sciences, Faculty of Health Sciences, The University of Bamenda, P. O Box, 818 Bamenda, Cameroon; 2 Bamenda Regional Hospital, Bamenda, Cameroon; 3 Department of Internal Medicine and Paediatrics, Faculty of Health Sciences, The University of Buea, Buea, Cameroon; 4 Buea Regional Hospital, Buea, Cameroon; 5 Department of Internal Medicine and Specialties, Faculty of Medicine and Biomedical Sciences, The University of Yaounde I, Yaounde, Cameroon; 6 Yaounde General Hospital, Yaounde, Cameroon

**Keywords:** Complementary and alternative medicine (CAM), Haemodialysis, Prevalence, Cameroon

## Abstract

**Background:**

Despite progress in haemodialysis and conventional medicine approaches, many patients still struggle to maintain an acceptable quality of life and turn to complementary and alternative medicine (CAM) to address their unmet needs.

**Objective:**

This study aims to determine the prevalence, types, indications, and factors associated with CAM use by patients on maintenance haemodialysis (MHD) in Cameroon.

**Materials and methods:**

This was a multicentric cross-sectional study involving MHD patients in Cameroon from February 2019 to May 2019. We included all consenting participants on MHD for at least 3 months and excluded participants with cognitive and behavioral problems. Face-to-face interviews were conducted.

**Results:**

A total of 224 participants (145 males) with a mean age of 56.5 ± 14.2 years and a median haemodialysis vintage of 34.5 [IQR: 17.3–64.4] months were recruited. In all, 89.7% (*n* = 201) reported having used CAM before, while 71.6% (*n* = 144) were still using it. Biologically based therapies were the most popular (94%, *n* = 189), with herbal medicine (81.5%, *n* = 154) and Calabar chalk (52.4%, *n* = 99) being the most common. Physical well-being (57.2%), nausea (52%), and insomnia (42.7%) were the main indications for CAM use. Most respondents did not disclose their CAM use to their physicians (61.2%). Long haemodialysis vintage was associated with CAM use (AOR: 7.9; CI = 2.8–22.3; *p* < 0.001).

**Conclusions:**

The use of CAM is common among Cameroon’s haemodialysis population, with herbal medicines and Calabar chalk being the most frequent. The high symptom burden makes CAM attractive to them. Healthcare teams should be aware of these practices, initiate an open discussion, and appropriately advise patients about dangers, risks, and safety associated with their use.

**Trial registration:**

The institutional review board of the University of Bamenda. Reference: 2019/0038H/UBa/IRB UPM/TNCPI/RMC/1.4.18.2.

**Supplementary Information:**

The online version contains supplementary material available at 10.1186/s12906-022-03815-7.

## Introduction

Chronic kidney disease is a major global health problem [1]. In advanced cases, it is associated with high mortality and morbidity, poor quality of life, and high resource utilization and cost of care [2]. Despite significant medical advances over several decades in the management of end-stage kidney failure (ESKF), these patients still face a multitude of physical and emotional symptoms and exhibit substantial impairment in quality of lif e[3]. To control and cope with their symptoms and improve their quality of life, many turn to complementary and alternative medicine (CAM). However, the use of CAM among this subpopulation is of great concern, as biologically based therapies which are the most widely used CAM modalities worldwide, seem to pose a greater risk to this population than to the general population [4]. This is mainly due to the poor excretory function that could alter pharmacokinetics and pharmacodynamics leading to increased toxicity, a greater chance of drug interactions, and potentially lethal electrolyte abnormalities [5].

CAM is defined as any medical and health care systems, practices, or products that are not thought of as standard medical care [6]. There is increasing use of CAM worldwide particularly in low- and middle-income countries, with claims of its benefits and non-nocive nature compared to conventional medicine [4]. The reported global prevalence of CAM use in the general population ranges from 10 to 94% with wide variation due to factors such as differences in the population characteristics, response rates, CAM definitions used, and study methodology. In European countries, the prevalence ranges between 10 and 40% [7], 40–60% in the USA [8], 63.1% in Australia [9], 62.5%.in Saudi Arabia [10] and 4.6–94% in sub-Saharan Africa [11]. In Cameroon, 80% of the population uses CAM mostly based on plants [12].

Data on this practice among the ESKF population worldwide remain scarce possibly because many patients who use CAM withhold this information from their primary healthcare team [13]. Previous worldwide studies revealed a prevalence of CAM use among haemodialysis patients ranging between 18.8 and 67% [14–20]. The modalities and sources of these forms of CAM vary around the world depending on cultural or traditional beliefs. The aim of this study was to determine the prevalence, modalities, and factors associated with CAM use among maintenance haemodialysis patients in Cameroon.

## Materials and methods

### Study design and setting

This multicentric cross-sectional study was conducted over a period of 4 months from February 2019 to May 2019 in Cameroon. Three state-owned haemodialysis centres were selected (convenience): the Yaounde General Hospital (YGH), Bamenda Regional Hospital (BaRH), and the Buea Regional Hospital (BRH) haemodialysis centres. The Bamenda and Buea regional hospitals are second-level hospitals located in the North and South West regions of the country, respectively, whereas the Yaounde General Hospital is a fourth-level hospital located in the capital city of Cameroon, precisely in the Center region. All three facilities are government funded and offer a twice-weekly dialysis program of 8 h per week. The 3 centers are all managed by nephrologists.

In Cameroon, out-of-pocket payments account for the majority of health-care financing. Since 2002, the government has subsidized haemodialysis sessions in public-sector centres. Patients are charged XAF 5000 ($ 7.79) per haemodialysis session. This price excludes the costs of vascular access, laboratory tests, medication, feeding, transportation, hospitalization, and vaccination. All of these extra expenditures are borne by patients and their families.

### Study participants and procedure

We included consenting participants on maintenance hemodialysis (MHD) for at least 3 months and excluded patients with cognitive impairment or cancer and those unable to communicate verbally or to complete the interview. Patients were met on the day of their dialysis and at the dialysis center. Face-to-face interviews using a questionnaire were conducted either during the haemodialysis session or in the waiting room.

### Sample size calculation

Sampling was consecutive and exhaustive. The sample size estimation was based on Bahall’s prevalence in Trinidad [14], and the online Sample Size Calculator software was used. The minimum sample required was *n* = 167 to have a confidence level of 90% and a margin of error of 5% of the surveyed value.

### Questionnaire

A prestructure questionnaire was designed for this study. This questionnaire contains 2 main sections: a sociodemographic and clinical section and a section on CAM use. The patient’s case file was used to complete the section pertaining to the sociodemographic and clinical information. For the cultural area of origin, participants specified whether they were from grassfield (found in the northwestern and western regions), soudano-Sahel (found in the Far-North, North and Adamawa regions), coastal (found in the littoral and southwestern regions), forest (found in the Central and southern regions) backgrounds or nonnationals. Other sociodemographic and clinical information collected included age, gender (male and female), religion (Christian, Muslim, atheist, and other), marital status (single, married, divorced, and widowed), level of formal education (none, primary, secondary and tertiary), comorbidities, aetiology of ESKF, actual treatment and duration of haemodialysis. For the use of CAM, we were interested in the nature and forms of CAM used, the sources of information and procurement, the reason for utilization, and disclosure of CAM use to the treating physician.

### Variables

Independent variables were sociodemographic characteristics (age, sex, marital and educational status), the presence of comorbidities, aetiologies of ESKF, duration of haemodialysis, use of CAM, modalities of CAM used, reasons for CAM use, and disclosure of CAM use to the treating physician. Dependent variables were the prevalence of CAM use, and factors associated with CAM use among haemodialysis patients.

### Data collection

The data on patient sociodemographics and disease-related information were collected from the patient’s case files, while the knowledge of CAM and its use by the participants were assessed using a face-to-face interview. All cases were identified by the code number. The information collected was based on the PROFORMA checklist. These data were entered into the Census Survey Processus (CSPro version 7.2) system.

### Data analysis

Data entered into the CSPro version 7.2 system were exported to the Statistical Package for Social Sciences (SPSS) version 23.0 software for statistical analysis (descriptive and inferential analysis). The descriptive methods included frequency distribution, tables, and graphs. Binary logistic regression was used to identify the predictors of CAM use in the study population based on significant associations identified from sociodemographic variables. All hypotheses were tested at the 5% level of significance. Analysed data are presented as odds ratios (ORs), 95% confidence intervals (95% CIs), and *p*-values. For multiple logistic regression, only variables with p-values < 0.20 or any clinically significant factor were selected for multiple logistic regression analysis.

### Definition of terms

Complementary and alternative medicine (CAM) was defined as any health-related practice that was not prescribed by a medical doctor and not considered conventional medicine.

The modalities of CAM therapies are classified into four categories as in Zakaria et al’s study [4]. These are divided into biologically based therapies, manipulative body-based therapies, mind-body interventions, and alternative medical systems. Biologically based therapies included plants and plant extracts (garlic, ginseng, *aloe vera*, moringa, green tea, ginger, guava leaves,etc.), geophagic naturally occurring minerals (Calabar chalk), dietary supplements (vitamins B and D) and animal-derived products (honey, eggs). Herbs are defined as any plant part that is used as medicine in any form (leaves, stems, roots, herbal teas, fruits except fruit juices). We are considered as manipulative and body-based therapies, the scarification and massage. Meditation, prayer, fasting, anointed water/oil/bracelets/stickers/cross, sacrifices, music therapy, holy water, exorcism, and burning of incense were considered mind and body interventions. We defined music therapy as regular, intentional listening to music for relaxation and general well-being. We considered as folk/indigenous medicine, the magic, divination, and sacrifices to deities done for health purposes. The level of education was divided into 4 categories: none (for those who had never been to school), primary (for those who interrupted their primary schooling), secondary (for those who interrupted their secondary schooling) and tertiary (for those with university studies).

## Results

**Fig. 1:**
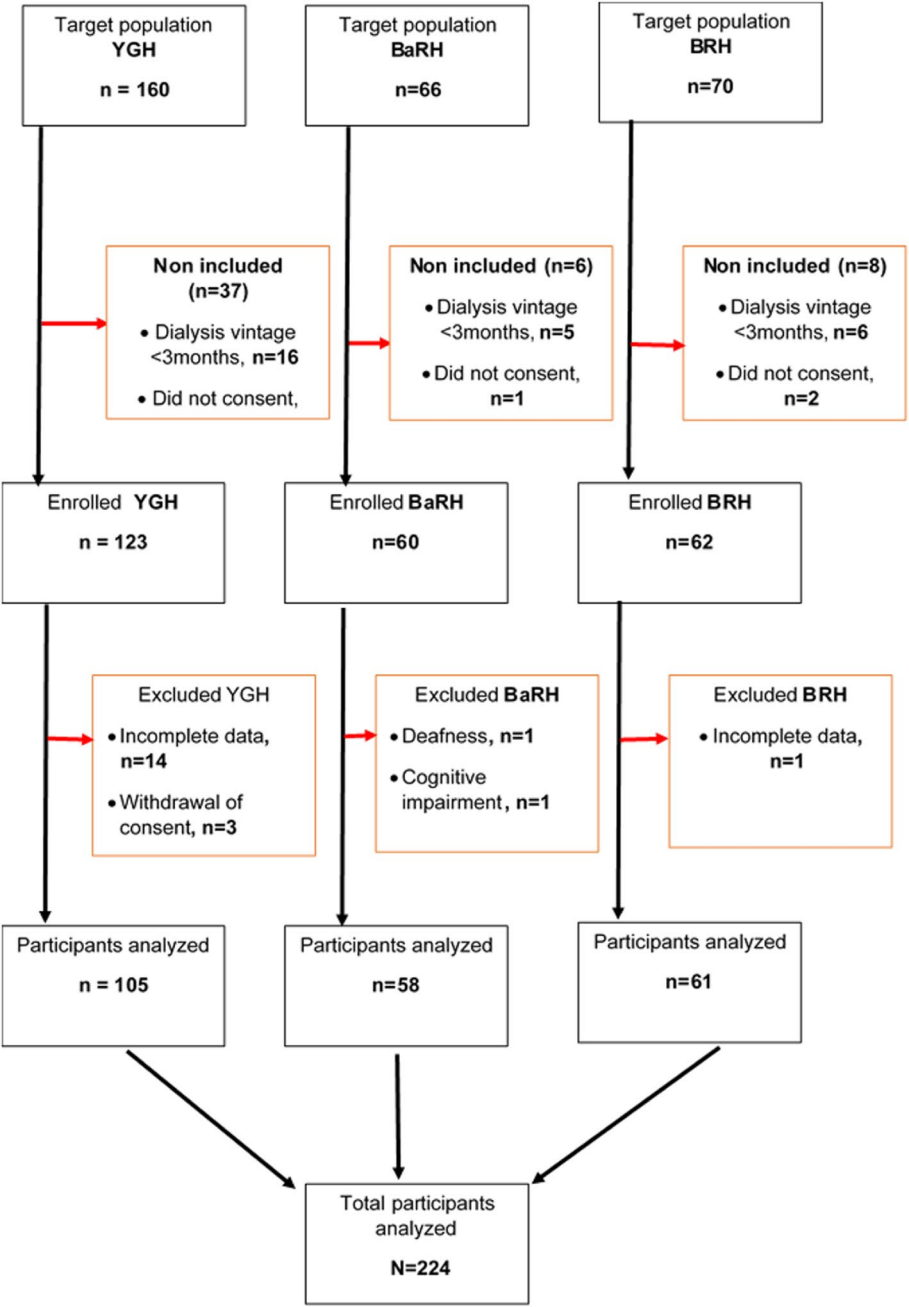
Recruitment flow chart. YGH: Yaounde General Hospital; BaRH: Bamenda Regional Hospital; BRH: Buea Regional Hospital

A total of 296 patients were approached. Among these patients, 72 were excluded, 27 patients for a haemodialysis vintage of fewer than 3 months, 27 patients for no consent, 15 for an incomplete interview, 2 patients had cognitive impairment, and 1 was deaf-and-dumb (see Fig. 1). A total of 224 participants were thus interviewed (145 males), with a mean age ± SD of 56.5 ± 14.2 years and a median haemodialysis vintage of 34.5 [17.4–64.4] months. Most of the participants were from grassfields (54.0%), Christians (93.3%), married (61.2%; *n* = 137) and had received secondary education (77.2%; *n* = 173). A total of 105 (48.8%) participants were from the YGH, 61 (27.2%) from the BRH, and 58 (25.8%) from the BaRH. Table 1 summarizes the sociodemographic data. The main comorbidities of the participants were hypertension (83.9%; *n* = 188), diabetes mellitus (13.4%; *n* = 30) and HIV (8.9%; *n* = 20). The comorbidities and aetiologies of ESKF of the respondents are outlined in Table 2.

**Table 1:** Sociodemographic characteristics of participants (*N* = 224)

Variable	***N*** = 224	Yaounde ***N*** = 105	Bamenda ***N*** = 58	Buea ***N*** = 61
**Age**^a^ **(years)**	46.5 ± 14.2^a^	49.1 ± 14.2^a^	44.1 ± 14.2^a^	44.3 ± 13.9^a^
**The cultural area of origin n (%)**				
Grass field	121(54.0)	38(36.2)	54(93.1)	31(50.8)
Forest	52(23.2)	49(46.7)	2(3.4)	1(1.6)
Coastal	38(17.0)	10(9.5)	1(1.7)	27(44.3)
Soudano Sahel	11(4.9)	8(7.6)	1(1.7)	2(3.3)
Nonnationals	2(9.0)	2		
**Religion n (%)**				
Christian	209(93.3)	96(91.4)	55(94.8)	59(96.7)
Muslim	14(6.3)	9(8.6)	3(5.2)	2(3.3)
atheist	1(4.0)			
**Level of formal education n (%)**				
None	16(7.1)	6(5.7)	9(15.5)	1(1.6)
Primary	35(15.6)	13(12.4)	7(12.1)	15(24.6)
Secondary	93(41.5)	47(44.8)	21(36.2)	25(41.0)
Tertiary	80(37.5)	39(37.1)	21(36.2)	20(32.8)
**Marital status n (%)**				
Single	87(38.8)	42(40.0)	20 (34.5)	25(41.0)
Married	137(61.2)	63(60.0)	38(65.5)	36(50.9)

^a^ mean ± standard deviation

**Table 2:** Frequency of comorbidities, HD vintage, and etiology of ESKF according to dialysis centers(*N* = 224)

Variable	***N*** = 224	Yaounde ***N*** = 105	Bamenda ***N*** = 58	Buea ***N*** = 61	
**Comorbidity**					
Hypertension n(%)	188(83.9)	82(78.1)	52(89.7)	54(88.5)	
Diabetes mellitus n(%)	30(13.4)	14(13.3)	3(5.2)	13(21.3)	
HIV n(%)	20 (8.9)	7(6.7)	6(10.4)	7(11.5)	
Hepatitis C n(%)	16(7.1)	9(8.6)	2(3.4)	1(1.6)	
Hepatitis B n(%)	12 (5.4)	7(6.7)	7(12.1)	2(3.3)	
Physical disability n(%)	5 (2.2)	3(2.9)	0(0.0)	2(3.3)	
**Etiology of ESKF**					
Hypertension n(%)	86(38.4)	40(38.1)	28(48.3)	18(29.5)	
CGN n(%)	65(29.0)	24(22.9)	17(29.3)	24(39.3)	
Diabetes n(%)	19(8.5)	11(10.5)	2(3.4)	6(9.8)	
HIV n(%)	14(6.3)	4(3.8)	3(5.2)	7(11.5)	
Unknown n(%)	11 (4.9)	7(6.9)	2(3.6)	2(3.1)	
CIN n(%)	9(4.0)	5(4.8)	4(6.5)	0(0.0)	
ADPKD n(%)	9(4.0)	4(3.9)	4(6.9)	1(1.6)	
**HD vintage in months**^a^	^a^34.5 [17.3–64.4]	^a^38.6 [20.7–77.2]	^a^37.7 [19.1–77.5]	^a^28.9 [7.4–43.4]	

^a^median [interquartile range], *HIV* Human immunodeficiency virus, *ESKF* End-stage kidney failure, *CGN* Chronic glomerulonephritis, *CIN* Chronic interstitial nephritis, *ADPKD* Autosomal dominant polycystic kidney disease, *HD vintage* Haemodialysis vintage or duration of haemodialysis therapy

**Fig. 2:**
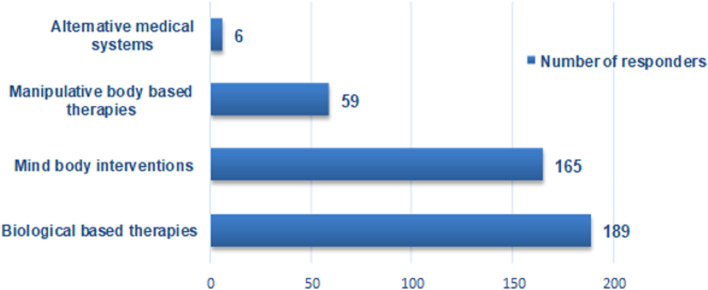
Number of CAM users based on the modalities of CAM use (some respondents with multiple responses)

**Fig. 3:**
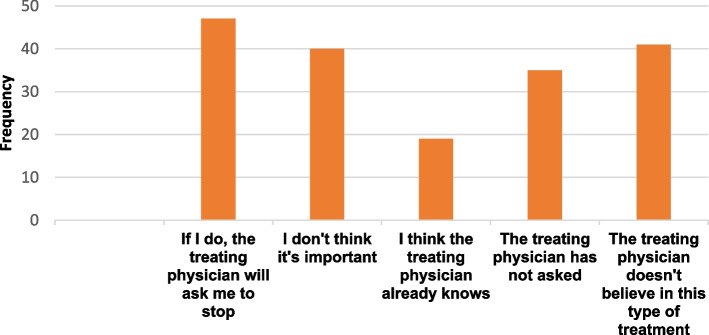
Reasons for nondisclosure of CAM use to treating physicians (*N* = 201)

Concerning CAM use, 89.7% (*n* = 201) of the participants reported having used CAM for the treatment of ESKF-related symptoms, with 71.6% (*n* = 144) reporting use within a month of the survey. Among the four categories of CAM, biological-based therapies (94%, *n* = 189) and mind-body practice (82.1%, *n* = 165) were the most popular CAMs used (see Fig. 2). The biological-based therapies were dominated by herbal medicine (81.5%; *n* = 154), and Calabar chalk (52.4%; *n* = 99) (see Table 3). Calabar chalk, also known as calabar stone, is a geophagic mineral stone belonging to the kaolinite family made up of fossilized seashell clay material. The mean number of herbs used per participant was 3.7 ± 2.5. Over half of the participants (63%, *n* = 97) used more than 2 herbs. Garlic (35.3%) and beetroot (27.4%) were the most frequently used herbs (see Table 4). Christian spiritual healing was the most common form of mind-body practice in 93.3% (*n* = 209) of our community. Family members and friends (67%) and other patients (42.3%) were the main sources of information about CAM (see Table 5). Home/farm (61.7%, *n* = 124) and market/supermarket (61.2%, *n* = 123) were the main sources of procurement. Physical well-being (57.2%), nausea (52%), and insomnia (42.7%) were the main reasons for CAM use (see Table 6). The majority (61.2%; n = 123) of participants did not disclose their CAM usage to their treating physicians, mainly because they assumed their physician would disapprove (38.2%, *n* = 47) or that the physician did not believe in it (33.3%, *n* = 41) (see Fig. 3).

**Table 3:** Types of biological-based therapy use (*N* = 189)

	Frequency	%
**Herbal medicine**	154	81.5
**Calabar chalk**	99	52.4
**Special diets**	82	43.4
Warm water	50	26.5
Sugar-free diet	35	18.5
Ice cold water	22	11.6
**Animal extracts and products**	81	42.9
Honey	79	41.8
Quail eggs	9	4.8
**Bitter kola (*Garcinia kola*)**	81	42.9
**Dietary supplements**	65	34.4
multivitamins	47	24.9
Omega 3	12	6.3
Clean shield	11	5.8
Alpha meta (amino acids, vitamins and minerals	10	5.3
Tre en en (omega 6, omega 3)	9	4.8
Calcium	6	3.2
**Other plant extracts**	24	12.7
Fruit juices	13	6.9
Apple cider vinegar	9	4.8

**Table 4:** Herbal medicine used by participants (*N* = 146)

Scientific (local)name	Frequency	%
Garlic (*Allium sativum*)	71	35.3
Beetroot (*Beta vulgaris*)	55	27.4
Herbal tea	52	25.9
Ginger (*Zingiber officinalis*)	47	23.4
Lemon grass (*Cymbopogon*)	41	20.0
*Aloe vera*	41	20.0
hibiscus flower (*Hibiscus rosa-sinensis*)	36	17.9
Moringa seeds (*Moringa oleifera*)	30	14.9
Ginseng (*Panax ginseng*)	21	10.4
Onions (*Allium cepa*)	20	10.0
Parsley (*Petroselinum crispum*)	18	9.0
Sour sop *(Annona muricate)*	14	7.0
Guava leaves (*Psidium guajava*)	13	6.5
Tomatoes (*Solanum lycopersicum)*	13	6.5
Two-sided leaves (*Anemomastax speciosa)*	12	6.0
Paw paw leaves (*Carica papaya)*	11	5.5
Masssopo (*ocimum gratissimum)*	10	5.0
Bitter leaves (*Vernonia*)	10	5.0
Blood leave (*Asystasia vogeliana*)	6	3.0
Okra (*Abelmoschus esculentus*)	5	2.5

**Table 5:** Distribution of participants according to the source of information about CAM (*n* = 201)

Variable	Frequency	Percentage
Family members and friends	134	66.7
Other patients	85	42.3
Advertisements/newspapers	38	18.9
Nontreating health personnel	27	13.4
Internet	23	11.4
TV/radio	9	4.5
Traditional healer	7	3.5
Church	3	1.5

**Table 6:** Distribution of respondents by indication/reasons of CAM use (*n* = 201)

Variable	Frequency	percentage
General well being	115	57.2
Nausea	105	52.2
Insomnia	86	42.7
Pain	80	40.0
Anaemia	76	37.8
Hypertension	65	32.3
Dyspepsia	47	23.4
Anorexia	45	22.4
Constipation	38	18.9
Muscle cramps	32	15.9
Fatigue	28	13.9
Cough	27	13.4
Uremic fetor	16	8.0
Diabetes	13	6.5
Vomiting	13	6.5
Diarrhea	10	5.0
Erectile dysfunction	8	4.0
Skin hyperpigmentation	6	3.0
Anuria	6	3.0
Pruritus	4	2.0

Long haemodialysis vintage (AOR: 7.9; CI = 2.8–22.3; *p* < 0.001) was the only factor independently associated with CAM use (see Tables 7 and 8).

**Table 7:** Factors associated with CAM use (Bivariate analysis)

Variables	CAM use		OR (95% CI)	***p***-value
	Yes	No		
**Age**				
> 40	131(65.2)	17(73.9)	0.7(0.2–1.8)	0.404
< 40	70(34.8)	6(26.1)	1	
**Sex**				
Male	129(89.0)	16(11.0)	0.8(0.3–1.9)	0.609
Female	72(91.1)	7(8.9)	1	
**The cultural area of origin**				
Grass field	112(55.7)	9(39.1)	1	**0.043**
Others	87(43.3)	14(60.8)	4.6(1.1–20.7)	
**Religion**				
Christian	189(94.0)	20(87.0)	1.9(0.6–6.7)	0.289
Muslim	11(5.5)	3(13.0)	1	
**Level of formal education**				
< secondary	46(22.9)	5(21.7)	0.9(0.3–2.7)	0.901
> secondary	155(77.1)	18(78.3)	1	
**Marital status**				
Married	124(61.7)	13(56.5)	0.8(0.3–1.9)	0.630
single	77(38.3)	10(43.5)	1	
**Hypertension**				
Yes	168(83.6)	20(87.0)	0.7(0.2–2.7)	0.677
no	33(16.4)	3(13.0)	1	
**Diabetes mellitus**				
Yes	27(13.4)	3(13.0)	1(0.3–3.7)	0.959
No	174(86.6)	20(87.0)	1	
**HD vintage in months**				
> 24	141(70.1)	6(26.1)	0.9(0.952–0.992)	0.006
< 24	60(29.9)	17(73.9)	1	

*Others* Soudano sahel (4.0%, *n* = 8), forest (22.4%, *n* = 45), coastal (16.9%, *n* = 34) *CAM* Complementary and alternative medicine, *OR* Odds ratio, *CI* Confidence interval

**Table 8:** Factors associated with CAM use (multivariate analysis) (*n* = 201)

Variable	AOR	95%CI	Adjusted p-value
HD vintage > 24(months)	7.9	2.8–22.3	< 0.001
Grass field	2.3	0.9–5.8	0.073

*AOR* Adjusted odds ratio, *CI* Confidence interval, *HD* Haemodialysis

## Discussion

This multicentric cross-sectional study documents the prevalence and pattern of CAM use in a haemodialysis population in Cameroon. We observed that 89.7% of subjects used CAM. The majority (61.2%) did so without informing their treating physician. Biological-based therapy (94%, *n* = 189) was the most commonly used form of CAM, followed by mind-body practices (82.1%, *n* = 165). A long duration on haemodialysis (> 24 months) was an independent predictor of CAM use.

We found a higher prevalence of CAM use in this study, greater than those previously described worldwide [14–20]. This high prevalence of CAM use in our setting probably reflects the high prevalence of CAM use in the general population. Indeed, the prevalence of CAM use varies according to the cultural beliefs and practices of different populations [19]. Among the general population in Cameroon, the prevalence of CAM use was reported to be 80%, similar to our results [12]. Many factors may explain this high prevalence. The high symptom burden, escalating comorbid illnesses, progressive decline in clinical state and quality of life, and absence of implementation of patient-centred palliative care in our haemodialysis centres cause high frustration and a loss of trust in conventional medicine. In addition, the high cost of conventional medical treatment and the cultural belief in the safety and efficiency of CAM compared with conventional medicine make CAMs attractive for these populations [11, 21, 22]. It is therefore not surprising that patients turn to CAM lightly to meet their physical, psychological, and spiritual needs, which are often undertreated by their physicians.

We observed that most of the CAM use was undisclosed to the treating physician, as has been reported previously [19, 20, 23]. The reasons for this attitude include the assumption that their physician would disapprove of it (38.2%) or that the physician did not believe in it (33.3%). It is therefore crucial for nephrologists to inquire about such practices, which could interact with medications, affect adherence to conventional therapy, and could be harmful [23].

Biological-based therapy was the most common CAM used by the respondents, with herbal medicines and calabar chalk being the most frequent. These findings are consistent with those described in the general population in Cameroon and sub-Saharan Africa, describing herbal medicines as the most commonly used form of CAM [11]. However, because of the unpredictable pharmacokinetics of these herbal medicine constituents in renal failure, their use is not always safe in ESKF. Moreover, most of these biological-based therapies were homemade remedies obtained from farms (61.7%) and local markets (61.2%) and were guided by nonmedical family members, friends, or other patients. Although some herbal medicines may have evidence supporting their use, some have no reliable evidence, and others may be potentially harmful [23–25]. Garlic (*Allium sativum*: 35.3%) was the most common herbal product used by our participants. Several studies have confirmed its effectiveness in lowering blood lipids, glucose, systolic blood pressure, inflammatory biomarkers, and platelet activity [26–28]. However, garlic has also been shown to interact with antihypertensive medications such as calcium channel blockers, which are commonly used by these patients. Garlic also has antiplatelet and anticoagulant properties, which may increase the risk of bleeding, particularly in patients taking heparin or aspirin [26]. Another frequently used biological agent was calabar chalk, also known as calabar stone, which is a mineral stone belonging to the kaolinite family made up of fossilized seashell clay material but can be prepared artificially by molding and heating mixed clay, mud, sand, wood, and even salt [3]. Calabar chalk geophagia is used to counteract some uremic symptoms such as nausea, hyperkalaemia, and mouth odor. It has been reported to contain several poisonous substances such as lead, arsenic, aluminum, and alpha lindane [29]. Calabar chalk has also been linked to hepatic sinusoidal enlargement, gastrointestinal damage, bone demineralization, nervous and brain damage, and anaemic effects in animals and humans [30, 31]. In addition to the poisonous substances whose adverse effects have thus far been reported, calabar chalk contains kaolin, which coats the gastrointestinal tract. Kaolin adsorbs drugs and other substances, including toxins, which, in addition to reducing their bioavailability, initiate diarrhoeal episodes, thus providing bulk to the stool [31, 32].

Mind-body practices (82.1%, *n* = 165) were the second most common CAM modality used in all 3 haemodialysis centres and were used concomitantly with other CAMs in most cases. This high frequency of its use in our population contrasts with low rates (4 to 42%) reported in other communities [14, 15, 20]. However, this may be due to differences in the definition of mind-body interventions used and differences in familiarity with this CAM modality. Some studies have suggested that mind-body practices may be useful for patients with ESKF [15, 33–35]. Mind-body interventions such as prayer, relaxation, or exorcism often involve self-care-based actions that are not expensive, produce relaxing effects, and can bring about beneficial physiological, functional, and psychological changes in MHD patients. They have almost no adverse effects and only rarely interact with conventional treatment [13]. Christian spiritual healing was the most common form of mind-body practice, as 93.3% (*n* = 209) of our community was Christians.

We found that a long duration of MHD (HD vintage > 24 months) was independently associated with CAM use on multivariate analysis (OR = 7.9; *p*-value < 0.001). A similar finding was observed in India [13] but was not confirmed in the USA [15]. Patient frustration with the efficacy of conventional medicine and/or the cost frequently explains this attraction to the CAM.

## Conclusion

The use of CAM is common among Cameroon’s haemodialysis population, with herbal medicines and Calabar chalk being the most frequent. The long haemodialysis vintage favours this practice. Many physicians are frequently not involved in the decision to use them. Healthcare teams should be aware of these practices, initiate an open discussion, and appropriately advise patients about the dangers and safety associated with their use.

## Study limitations

This study has some limitations. Its cross-sectional design renders the result subject to recall bias regarding CAM use. However, this was an exhaustive review of the subjects in all 3 haemodialysis centres, and to our knowledge, this is the first study carried out in Cameroon examining the use of CAM among this subpopulation.

## Supplementary Information

**Additional file 1.**

**Additional file 2.** .

## Data Availability

The datasets used and/or analysed during the current study are available from https://zenodo.org/record/7217860#.Y03X_XbMLrc

## Abbreviations

AOR: Adjusted odds ratio
BaRH: Bamenda regional hospital
BRH: Buea Regional Hospital
CAM: Complementary and alternative medicine
CGN: Chronic glomerulonephritis
ESKF: End stage kidney failure
MHD: Maintenance haemodialysis
USA: United States of America
YGH: Yaounde General Hospital
