# Supplementary material for: Frequency, types, and factors associated with complementary and alternative medicine use among patients on maintenance haemodialysis
Source: BMC Complement Med Ther. 2022 Dec 7;22:325. doi: 10.1186/s12906-022-03815-7 (PMC9727851; doi:10.1186/s12906-022-03815-7)
Supplement: Supplementary file 2 — Additional file 2. . [file 12906_2022_3815_MOESM2_ESM.pdf]

FREQUENCY, TYPES AND FACTORS ASSOCIATED WITH COMPLEMENTARY AND ALTERNATIVE MEDICINE USE AMONG PATIENTS ON MAINTENANCE HAEMODIALYSIS

QUESTIONNAIRE

site: YGH |___| BaRH |___| BRH |___|

SECTION 0: IDENTIFICATION

S0Q01	Participants code	

S0Q01 Participants code

S1Q01	**Date of birth** / / 19…..<br>Age…………..years	
S1Q02	**Sex**<br>1-Male 2-Female	|___|
S1Q03	**Religion**<br>1-Christian 2-Muslim 3-Atheist 4-Other(specify)	|___|
S1Q04	**If Christian, denomination**<br>1-catholic 2-protestant 3-pentecostant	|___|
S1Q05	**Cultural areas of origin** 1-grassfield 2- sahelian 3-coastal 4-forest 5- non nationals(specify)	|___|
S1Q06	**Level of formal education**<br>1- None 2- Primary 3- Secondary 4- Tertiary	|___|
S1Q07	**Marital Status**<br>1- Single 2- Married	|___|
S1Q08	**If married,** 1-monogamy 2-polygamy	|___|

SECTION 2: COMORBIDITIES

S2Q01	**HTN**: 1- Yes 2- No	|___|
S2Q02	**On treatment?** 1- yes 2- no	|___|
S2Q04	**Good blood pressure control** 1-yes 2-no	|___|
S2Q05	**Diabetes** 1- Yes 2- No	|___|
S2Q06	**Treatment type** 1- oral antidiabetic 2- insulin 3-diet only	|___|
S2Q07	**Good glycaemia control** 1-yes 2-no	|___|
S2Q08	**HIV**: 1- Yes 2- No	|___|
S2Q09	**If yes, on** 1- on cART 2-no cART	
S2Q10	**Hepatitis B** 1-yes 2-no	|___|
S2Q11	**Hepatitis C** 1-yes 2-no	|___|
S2Q12	**Physical disability** 1-yes 2-no	|___|

S2Q13	**If yes , paralyses** 1-yes 2-no	|___|
S2Q14	**Blindness** 1-yes 2-no	|___|
S2Q15	**Amputation** 1-yes 2-no	|___|

SECTION 3: ETIOLOGY OF CKD

S3Q01	**Hypertension** 1-yes 2-no	|___|
S3Q02	**Diabetes** 1-yes 2-no	|___|
S3Q03	**Chronic glumerulonephritis** 1-yes 2-no	|___|
S3Q04	**HIV** 1-yes 2-no	|___|
S3Q05	**Autosomal dominant polycystic kidney disease** 1-yes 2-no	|___|
S3Q06	**Chronic tubulointerstitial nephritis** 1-yes 2-no	|___|
S3Q07	**Unknown** 1-yes 2-no	|___|
S3Q08	**Others** 1 yes 2 no	|___|

SECTION 4: CLINICAL PARAMETERS

S4Q01	**Blood pressure** (mmHg)	SBP	
		DBP	
S4Q02	**Height**(m)		
S4Q03	**Weight**(kg)		
S4QO4	**BMI(kg/m2)**		

SECTION 5: HEMODIALYSIS DATA

S5Q01	**date of initiation of dialysis** **………. /………/** 20……..	

SECTION 6: THERAPEUTICS

S6Q01	**Calcium channel blocker** 1-yes 2-no	|___|
S6Q02	**Angiotensin receptor antagonist** 1-yes 2-no	|___|
S6Q03	**Sulfonylurea** 1-yes 2-no	|___|
S6Q04	**Biguanides** 1-yes 2-no	|___|
S6Q05	**Protease inhibitor** 1-yes 2-no	|___|
S6Q06	**NNRTI** **1**-yes 2-no	|___|
S6Q07	**ACE inhibitors** 1-yes 2-no	|___|
S6Q08	**aspirin** 1-yes 2-no	|___|
S6Q09	**Vit D** 1- yes 2- no	|___|
S6Q10	**Digoxin** 1-yes 2-no	|___|
S6Q11	**Vit B** 1- yes 2- no	|___|
S6Q12	**Statin** 1-yes 2-no	|___|
S6Q13	**Erythropoeitin** 1- yes 2- no	|___|
S6Q14	**Anti coagulant** 1-yes 2-no	|___|

SECTION 7: FREQUENCY OF CAM USE

S7Q01	**Apart from the treatment you receive in the hospital and drugs prescribed by your doctor, Have you used any other treatment since you started dialysis?** 1- yes 2- no	|___|

SECTION 8: PREVALENCE OF CAM USE

S8Q01	**Do you currently use this treatment?** 1-yes 2-no	|___|
	**If no, why did you stop using it?**	
S8Q02	my doctor asked me to stop 1-yes 2-no	|___|
S8Q03	It was not working 1-yes 2-no	|___|
S8Q04	It was too expensive 1-yes 2-no	|___|
S8Q05	It wasn't available anymore 1-yes 2-no	|___|
S8Q06	Its side effects 1-yes 2-no	|___|
S8Q07	0ther reason 1-yes 2-no	|___|

SECTION 9: TYPES OF CAM USE and INDICATIONS FOR USING CAM

S8Q01	**What treatment did you use or are you using?**<br>***Biological Based Therapies (leaves,roots,stems,animal extracts,earth,seeds*** **)** **Garlic** 1-yes 2-no	|___|
S8Q02	**Ginseng** 1-yes 2-no	|___|
S8Q03	**Ginger** 1-yes 2-no	|___|
S8Q04	**Guava leaves** 1-yes 2-no	|___|
S8Q05	**Bitter leaves** 1-yes 2-no	|___|
S8Q06	**Aleovera** 1-yes 2-no	|___|
S8Q07	**Calaba chalk/kaolin** 1-yes 2-no	|___|
S8Q08	**Lemon** 1-yes 2-no	|___|
S8Q09	**Quail eggs** 1-yes 2-no	|___|
S8Q10	**Honey** 1-yes 2-no	|___|
S8Q11	**Folere** 1-yes 2-no	|___|
S8Q12	**Apple cider vinegar** 1-yes 2-no	|___|
S8Q13	**Warm water** 1-yes 2-no	|___|
S8Q14	**Alcohol** 1-yes 2-no	|___|
S9Q15	**Cola nuts** 1-yes 2-no	|___|
S9Q16	**Bitter cola** 1-yes 2-no	|___|
S9Q17	**sugar free diet** 1-yes 2-no	|___|
S9Q18	**Ice cold water**	|___|
S9Q19	**Moringa seed** 1-yes 2-no	|___|
S9Q20	**Beetroot** 1-yes 2-no	|___|
S8Q21	**Sour sop** 1-yes 2-no	|___|
S8Q22	**Fever grass** 1-yes 2-no	|___|
	**Quail eggs 1 yes 2 no**	|___|
	**Garden eggs 1yes 2 no**	|___|
	**Garden egg leaves 1yes 2 no**	|___|
	**Paw paw leaves 1 yes 2 no**	|___|
	**Fruit juices 1yes 2 no**	|___|
	**Onions 1yes 2 no**	|___|
	**Celery 1 yes 2 no**	|___|
	**Two sided leave 1yes 2 no**	|___|
	**Okra 1 yes 2 no**	|___|

	Kitchen sooth 1 yes 2 no	|___|
	Cassava leaves 1 yes 2 no	|___|
	Tomatoes 1 yes 2 no	|___|
	Grenadine 1 yes 2 no	|___|
	King seed 1 yes 2 no	|___|
	Ocimumgratissimum 1 yes 2 no	|___|
	Lemon 1 yes 2 no	|___|
	Asystasia vogeliana(blood leave) 1 yes 2 no	|___|
	Enemomastax speciose(two sided leave) 1 yes 2 no	|___|
	**Medicinal teas**	
	power tea 1 yes 2 no	|___|
	Chai tea 1 yes 2 no	|___|
	Green tea 1 yes 2 no	|___|
	Moringa tea 1 yes 2 no	|___|
	Slim tea 1 yes 2 no	|___|
	**Dietary supplements**	
	Omega 3 1 yes 2 no	
	24/7 1 yes 2 no	|___|
	Alpha meta 1 yes 2 no	|___|
	Clean shield 1 yes 2 no	|___|
	tre en en 1 yes 2 no	|___|
	Fish oils 1 yes 2 no	|___|
	Long rich iron, zinc, calcium 1 yes 2 no	|___|
	Vitamins 1 yes 2 no	|___|

	**Alternative systems**	
	Bio-disk 1-yes 2-no	|___|
	Alkaline cup 1-yes 2-no	|___|
	Longrich shoes	|___|

	**What were/are you using it for?**	
S9Q15	Nausea 1-yes 2-no	|___|
S9Q16	Anorexia 1-yes 2-no	|___|
S9Q17	Vomiting 1-yes 2-no	|___|
S9Q18	Pain 1-yes 2-no	|___|
S9Q19	Insomnia 1-yes 2-no	|___|

S9Q20	Anemia 1-yes 2-no	|___|
S9Q21	diabetes 1-yes 2-no	|___|
S9Q22	Fatigue 1-yes 2-no	|___|
S9Q23	HTN 1-yes 2-no	|___|
S9Q24	To cure my kidneys 1-yes 2-no	|___|
S9Q25	Mouth odor 1-yes 2-no	|___|
S9Q26	It helps me urinate 1-yes 2-no	|___|
S9Q27	muscles cramps 1-yes 2-no	|___|
S9Q28	For general well being 1-yes 2-no	|___|
S9Q29	Darkened skin 1-yes 2-no	|___|
S9Q30	Diarrhea 1-yes 2-no	|___|
S9Q31	Easy digestion 1-yes 2-no	|___|
S9Q32	Cough 1-yes 2-no	|___|
S9q33	Constipation 1-yes 2-no	|___|
S10q34	For detoxification 1 yes 2 no	|___|
S11q35	Sexual weakness	|___|

	**How did you know about this treatment?**	
S9Q33	Medical doctor 1-yes 2-no	|___|
S9Q34	Nurse 1-yes 2-no	|___|
S9Q35	Dietician 1-yes 2-no	|___|
S9Q34	Family members and friends 1-yes 2-no	|___|
S9Q35	Traditional doctor/herbalist 1-yes 2-no	|___|
S9Q36	Internet 1-yes 2-no	|___|
S9Q37	TV/radio 1-yes 2-no	|___|
S9Q38	Other patient 1-yes 2-no	|___|
S9Q39	Church 1-yes 2-no	|___|
S9Q40	Advertisements 1-yes 2-no	|___|

	**Where did you get it from?**	
S9Q41	Home/farm 1-yes 2-no	|___|
S9Q42	Market /super market 1-yes 2-no	|___|
S9Q43	Traditional healer 1-yes 2-no	|___|
S9Q44	Sales agent 1-yes 2-no	|___|
S9Q45	Pharmacy 1-yes 2-no	|___|
S9Q46	Family member/friend 1-yes 2-no	|___|
S9Q47	Other patients 1-yes 2-no	|___|
S9Q48	Medical doctor 1-yes 2-no	|___|
S9Q49	Nurse 1-yes 2-no	|___|
S9Q50	Imported 1-yes 2-no	|___|

**Manipulative and body based therapies**		
S9Q49	Massage 1-yes 2-no	|___|

S9Q50	Scarifications 1-yes 2-no	|___|
	**What were/are you using it for?**	
S9Q51	Anxiety 1-yes 2-no	|___|
S9Q52	Depression 1-yes 2-no	|___|
S9Q53	Muscle cramps 1-yes 2-no	|___|
S9Q54	Pain 1-yes 2-no	|___|
S9Q55		|___|
		|___|
		|___|
		|___|
	**How did you know about this treatment?**	
S9Q56	Health care provider 1-yes 2-no	|___|
S9Q57	Traditional healer 1-yes 2-no	|___|
S9Q58	family members and friends/family history and tradition 1-yes 2-no	|___|
S9Q59	Other patients 1-yes 2-no	|___|
S9Q60	TV/Radio 1-yes 2-no	|___|
S9Q61	Internet 1-yes 2-no	|___|
	**Where did/do you get this service from?**	
S9Q62	Massage therapist 1-yes 2-no	|___|
S9Q63	Traditional healer 1-yes 2-no	|___|
S9Q64	Family members and friends 1-yes 2-no	|___|
S9Q65	I do it myself 1-yes 2-no	|___|
	**Mind and body interventions**	
S9Q66	Meditation 1-yes 2-no	|___|
S9Q67	Sacrifices 1-yes 2-no	|___|
S9Q68	Fasting 1-yes 2-no	|___|
	Prayers 1yes 2 no	|___|
S9Q69	Burning of incense 1-yes 2-no	|___|
S9Q70	holy or Anointed water/oil/sticker/bracelet/cross 1-yes 2-no	|___|
S9Q71	Music therapy 1-yes 2-no	|___|
S9Q72	Country fashion 1-yes 2-no	|___|
S9Q73	Walking 1-yes 2-no	|___|
S9Q74	Novena 1-yes 2-no	|___|
S9Q75	Exorcism 1-yes 2-no	|___|
	Exercise 1 yes 2 no	
		|___|
		|___|
**Have you discussed the use of this other treatments with your treating physitian?** 1-yes 2-no |___|		
	**If no, why?**	
S9Q84	If I do, he may ask me to stop 1-yes 2-no	|___|
S9Q85	I don’t think its important 1-yes 2-no	|___|
S9Q86	I think he already knows 1-yes 2-no	|___|
S9Q87	He has not asked 1-yes 2-no	|___|
S9Q88	He doesn’t believe in this other forms of treatment 1-yes 2-no	|___|

S9Q89	**Will you recommend any of these treatments to other patients?**	1-yes 2-no	|___|
